# Risk Factors for Catheter-Related Thrombosis in Multiple Myeloma Patients Undergoing Autologous Stem Cell Transplantation

**DOI:** 10.3390/medicina57101020

**Published:** 2021-09-26

**Authors:** Anna Hoppe, Joanna Rupa-Matysek, Bartosz Małecki, Dominik Dytfeld, Krzysztof Hoppe, Lidia Gil

**Affiliations:** 1 Department of Hematology and Bone Marrow Transplantation, Poznan University of Medical Sciences, 60-569 Poznan, Poland; rupa.matysek@gmail.com (J.R.-M.); bartosz.malecki@skpp.edu.pl (B.M.); dominik.dytfeld@skpp.edu.pl (D.D.); lidia.gil@skpp.edu.pl (L.G.); 2 Department of Nephrology, Transplantology and Internal Diseases, Poznan University of Medical Sciences, 60-355 Poznan, Poland; khoppe@ump.edu.pl

**Keywords:** catheter-related thrombosis, multiple myeloma, autologous stem cell transplantation

## Abstract

*Background and Objectives*: Cancer associated thrombosis (CAT) is a common complication of neoplasms. Multiple myeloma (MM) carries one of the highest risks of CAT, especially in the early phases of treatment. Autologous stem cell transplantation (ASCT) as the standard of care in transplant-eligible patients with MM carries a risk of catheter-related thrombosis (CRT). The aim of this study was identification of the risk factors of CRT in MM patients undergoing ASCT in 2009–2019. *Materials and Methods*: We retrospectively analyzed patients with MM undergoing ASCT. Each patient had central venous catheter (CVC) insertion before the procedure. The clinical symptoms of CRT (edema, redness, pain in the CVC insertion area) were confirmed with Doppler ultrasound examination. We examined the impacts of four groups of factors on CRT development: (1) patient-related: age, gender, Body Mass Index (BMI), obesity, Charlson comorbidity index, hematopoietic stem cell transplantation comorbidity index, renal insufficiency, and previous thrombotic history; (2) disease-related: monoclonal protein type, stage of the disease according to Salmon–Durie and International Staging System, number of prior therapy lines, and MM response before ASCT; (3) treatment-related: melphalan dose, transplant-related complications, and duration of post-ASCT neutropenia; (4) CVC-related: location, time from placement to removal. *Results*: Symptomatic CRT was present in 2.5% (7/276) of patients. Univariate analysis showed an increased risk of CRT in patients with a catheter-related infection (OR 2.4, 95% CI; 1.109–5.19, *p* = 0.026), previous thrombotic episode (OR 2.49, 95% CI; 1.15–5.39, *p* = 0.021), previous thrombotic episode on initial myeloma treatment (OR 2.75, 95% CI; 1.15–6.53, *p* = 0.022), and gastrointestinal complications of ASCT such as vomiting and diarrhea (OR 3.87, 95% CI; 1.57–9.53, *p* = 0.003). In multivariate analysis, noninfectious complications were associated with higher CRT incidence (OR 2.75, 95% CI; 1.10–6.19, *p* = 0.031). *Conclusions*: The incidence of symptomatic CRT in ASCT in MM was relatively low. Previous thrombotic events, especially during the induction of myeloma treatment, increased CRT risk during ASCT. Dehydration following gastrointestinal complications may predispose to higher CRT incidence.

## 1. Introduction

Thromboembolism is a common complication of cancer therapy. From among the various neoplasmatic disorders, multiple myeloma (MM) carries one of the highest risks of cancer-associated thrombosis (CAT) [1]. For MM patients, the risk of thromboembolic events is 9.2 times higher in comparison to healthy coevals [2]. The highest risk of CAT is within the first year after diagnosis, but it still remains higher beyond this period [2]. To date, multiple risk factors for CAT in MM have been identified, one of these is the presence of a central venous catheter (CVC) [3]. Despite novel therapies in MM, high-dose melphalan treatment followed by autologous stem cell transplantation (ASCT) remains the standard of care in transplant eligible myeloma patients [4]. An integral part of this process is the insertion of a central venous catheter before the procedure. In the majority of patients, tunneled CVCs are used [5,6]. Some groups of patients have implanted port-a-caths, peripherally inserted central catheters (PICCs) [7] or short-term non-tunneled CVCs [6]. In hematological patients, CVC insertion carries a risk of catheter-related thrombosis (CRT) of up to 34% [8,9]. The postulated mechanisms of CRT development are direct endothelial damage by catheter insertion, impaired blood flow through the blood vessel caused by the catheter itself or infused fluids, such as chemotherapy and other drugs, restriction of limb movement, and a hypercoagulable state in malignancy, sepsis, or local inflammation [10,11]. According to the literature, in the general population of patients, some factors that increase CRT risk have been evaluated. A hypercoagulable state induced by sepsis, malignancy, or critical illness; inherited thrombophilia; a previous venous thrombotic event (VTE); certain drug usage; PICCs; large lumen catheters, with the tip located above the junction between the SVC and atrium, left-sided and femoral access, multiple insertion attempts, and time from placement to removal, all seem to increase the thrombosis risk [12]. However, data on the risk factors of CRT in MM patients undergoing ASCT are scarce. The aim of the study was to identify the risk factors of CRT in MM patients undergoing ASCT.

## 2. Materials and Methods

### 2.1. Group Characterization

We retrospectively analyzed patients with MM undergoing ASCT in the Department of Hematology and Bone Marrow Transplantation of Poznan University of Medical Sciences between 2009 and 2019. All of them had a CVC inserted before the procedure. The study inclusion criteria were age ≥18 years, diagnosis of MM according to the International Myeloma Working Group [13], and treatment with ASCT. Patients receiving long-term anticoagulation due to the fact of recent thrombosis or cardiac arrythmia and patients on antiplatelet therapy were excluded from the analysis. Patients receiving immunomodulating drugs (IMIDs) and, thus, requiring thrombo-prophylaxis had IMIDs withdrawn at least 30 days before ASCT. All ASCT recipients underwent insertion of a CVC. All of the patients had short-term, non-tunneled triple-lumen catheters inserted via one of the central veins—internal jugular vein, subclavian vein, or femoral vein—prior to the start of the conditioning regimen. Transplant conditioning consisted of melphalan once on day −1. The dosage depended on the physician’s discretion: a full dose of 200 mg/m^2^ was administered in individuals with no or minor comorbidities, whereas subjects with higher Charlson comorbidity indices (CCIs) or transplant risk assessed in hematopoietic cell transplantation comorbidity indices (HCT-CIs) received a reduced dose of 140 mg/m^2^. For shortening of the neutropenic period and reduction of the infection risk, patients received granulocyte-colony stimulating factor (G-CSF). All participants were observed from the date of CVC insertion until removal due to the CRT or end of follow up (100 days after ASCT). Due to the local policy, patients received no thromboprophylaxis. All subjects developed severe thrombocytopenia (platelet count below 20 G/L) and required platelet concentrate transfusion. After platelet recovery (defined as platelet count > 40 G/L for 2 consecutive days), we introduced thromboprophylaxis with enoxaparin (40 mg daily). To identify CRT, we searched the hospital discharge diagnoses and the radiology procedure registry. A Doppler ultrasound (GE Voluson 730 Pro) examination was performed in the case of the onset of CRT symptoms (pain, redness, swelling on CVC insertion area, limb edema, dysfunction of CVC). CRT was defined as a partial or complete occlusion by thrombus of the blood vessel in which the catheter was present. We analyzed the influence on CRT development in MM patients undergoing ASCT using four categories of factors: (1) patient-related: age, gender, CCI, HCT-CI, previous thrombotic history, number of thrombotic episodes, thrombotic episodes before ASCT and post-ASCT, and thrombotic episodes during initial treatment of MM; (2) disease-related: Durie–Salmon Stage (DSS), International Staging System (ISS), and renal insufficiency before ASCT defined as glomerular filtration rate (GFR) according to the Modification of Diet in Renal Disease (MDRD) equation < 60 mL/min/1.73 m^2^; (3) treatment-related factors: conditioning regimen (melphalan full dose 200 mg/m^2^/reduced dose), complications:infectious (including CVC-related infection), noninfectious (gastrointestinal tract (GI) and cardiovascular system (CVS) complications), and neutropenia period duration; (4) catheter-related factors: location (left/right side, subclavian/jugular/femoral vein) and time from placement to removal of CVC. All patients diagnosed with CRT received treatment with therapeutic doses of enoxaparin (1mg/kg body weight twice a day or 1.5 mg/kg body weight daily).

### 2.2. Statistical Analysis

We used descriptive statistics for patients and disease characterization. The Shapiro–Wilk test was performed to assess normality. Statistical comparison was performed using the χ^2^ test with Yates’s correction and NW tests when required for categorical variables and the Mann–Whitney U test for continuous variables. Assuming a CRT rate of approximately 3% based on averages from literature [5,14], we calculated that at least 112 patients would be required for an alpha level of 0.05. The parameters that exerted any influence on CRT events were indicated by logistic regression. Multivariate logistic regression was used to evaluate potential risk factors that might influence CRT. In each model, the odds ratio (OR) for each independent variable was determined with a confidence interval (CI) of 95%. A *p*-value below 0.05 was regarded as statistically significant. All statistical analyses were performed using StatSoft Statistica version 13.0.

## 3. Results

### 3.1. Patient and CRT Cases Characteristics

In the study, we enrolled 276 patients with MM undergoing ASCT. The group characterization is described in Table 1. In the entire cohort, during follow up, symptomatic CRT was present in 2.5% (*n* = 7) of patients. The median age at the time of CRT was 63 years; 57% were male. The main demographic summary of CRT cases and catheter characteristics are shown in Table 2. Of the seven cases of CRT, six occurred during hospitalization. The CVC insertion sites were as follows: the right internal jugular vein—57%, right subclavian vein—29%, and femoral vein—14%. In all cases, CRT occurred with right side access. In the patients with CRT, the median time from CVC placement to CRT was 14 days (range, 11–26), while the median time from ASCT to CRT was 10 days (range 7–28). Fifty-seven percent of CRT patients were neutropenic during CRT onset. In the CRT group, 86% (*n* = 6) were thrombocytopenic. The median platelet count at the CRT diagnosis was 32 G/L (range 8–324G/L). The majority of patients had the tunneled line removed on the same day as CRT was identified, and the median time from CVC insertion to removal was 14 days (range 11–26 days).

### 3.2. Risk Factors for CRT and Transplant Outcomes

#### 3.2.1. Patient-Related Factors

In the entire cohort we identified 46 patients with a history of thrombotic episodes (TEs) other than CRT during ASCT, 41 of these developed one TE whereas five patients developed two TEs. We identified 34 TEs in 32 patients prior to ASCT. The most common TE was lower limb deep vein thrombosis—70% (24), two patients developed pulmonary thromboembolism (PE), CRT was present in 18% (6), other TEs 6% (2). Post-ASCT, 15 patients developed 17 TEs: 71% (12) lower limb deep vein thrombosis, 29% (5) PE.

**Figure 1:**
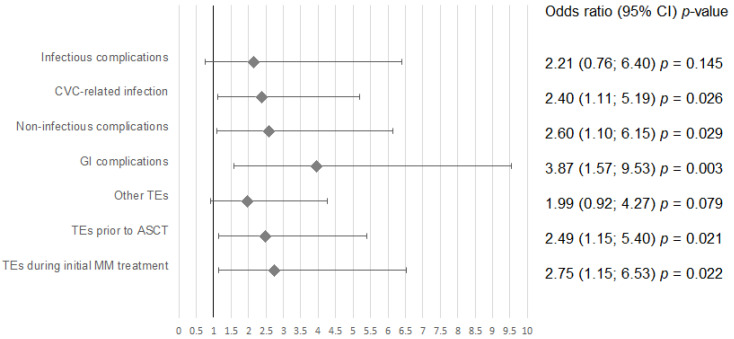
Univariate analysis for risk factors of catheter-related thrombosis. Abbreviations: CVC—central venous catheter, GI– gastrointestinal, TE– thromboembolic event, ASCT—autologous stem cell transplantation, MM—multiple myeloma.

Univariate analysis showed an increased risk of CRT in patients with previous thrombotic episodes before ASCT (OR = 2.49, 95% CI; 1.15–5.4, *p* = 0.021), previous thrombotic episodes during initial MM treatment (OR = 2.7, 95% CI; 1.15–6.53, *p* = 0.022), and number of thrombotic episodes (OR = 2.75, 95% CI; 1.01–4.7, *p* = 0.047). The results are presented in Figure 1.

Other patient-related factors (age, gender, BMI, CI, HCT-CI, and chronic kidney disease) have no impact on CRT incidence. The results are shown in Table 2.

#### 3.2.2. Disease-Related Factors

Eighty-eight percent of patients had at most two induction treatment regimens, while 66% required only one before ASCT. The regimen choice depended on physician discretion. Ninety-five percent of the patients received IMIDs. The most popular in Poland is a three-drug regimen composed of bortezomib, thalidomide, and dexamethasone; thus, in 65% (*n* = 178), this drug was introduced. Eighteen percent of patients (*n* = 50) received anthracyclines in a multidrug combination. None of the analyzed disease-related factors (M-protein type, Durie–Salmon stage (DSS), International Staging System (ISS), number of prior MM therapy lines, and response of MM before ASCT) were associated with a higher CRT risk. We found no significant difference for IMID usage before ASCT (*p* = 0.8).

#### 3.2.3. Treatment-Related Factors

Infectious complications were observed in 55% (*n* = 155) of patients. The most common was neutropenic fever without microbiological identification (63%; *n* = 97). A positive blood culture was present in 28% (*n* = 44), Gram-positive and Gram-negative bacteria were identified in 20% (*n* = 31) and 8% (*n* = 13) of patients with infectious complications, respectively. Twelve percent (*n* = 34) of patients presented catheter-related infection (CRI). The major cause (77%; *n* = 26) was Staphylococcus epidermidis, while other Gram-positive bacteria (Staphylococcus hominis and Staphylococcus aureus) and Gram-negative bacteria (Klebsiella pneumoniae, Proteus mirabilis, and Pseudomonas aeruginosa) were identified, respectively, in 9% (3) and 9% (3) of CRI cases. A catheter-related infection increased the risk of CRT (OR = 2.4, 95% CI; 1.11–5.19, *p* = 0.026). Septic shock, pneumonia, and Closterioides difficile infection represented, respectively, 4% (6), 2% (3), and 3% (4) of infectious complications.

Noninfectious complications developed in 6% (17) of patients and were associated with a higher CRT risk (OR = 2.60, 95% CI; 1.10–6.15, *p* = 0.029). Cardiac arrythmias present in 2% (5) had no impact on CRT risk, but gastrointestinal tract complications (noninfectious diarrhea and excessive vomiting) affecting 4% (9) of patients significantly increased CRT risk (OR = 3.87, 95% CI; 1.57–9.53, *p* = 0.003).

#### 3.2.4. Catheter-Related Factors

In the entire cohort, short-term, non-tunneled CVCs were used. The most popular access was via internal jugular vein (57%). In 57% (157) of the general population, a right-side insertion was performed. All patients from the CRT group had right-sided CVC, but we did not observe any statistical significance of right-side placement on CRT development.

#### 3.2.5. Multivariate Analysis

In multivariate analysis, catheter-related infection (OR = 2.78, 95% CI; 1.21–6.39, *p* = 0.016), previous thrombotic episode before ASCT (OR = 2.88, 95% CI; 1.24–6.70, *p* = 0.014) and noninfectious complications (OR = 2.75, 95% CI; 1.09–6.91, *p* = 0.031) were all associated with a higher CRT incidence. The results are presented in Table 3.

#### 3.2.6. Transplant Outcomes

In our study, CRT did not have any impact on patients’ long-term outcomes. Both groups, the CRT and the non-CRT group, had similar progression-free survival and overall survival.

## 4. Discussion

CVC insertion is an integral part of ASCT necessary for conditioning chemotherapy administration and hematopoietic stem cell transfusion. Moreover, it enables the provision of high-quality supportive treatment during the procedure: blood sampling, drug administration, blood compounds transfusion, and parenteral nutrition when required [15]. Widespread usage of CVC carries a risk of various complications: mechanical insertion-associated complications, such as pneumothorax, accidental arterial puncture, hematoma, and long-term complications such as thrombosis and catheter-related infections [15,16]. Notably, for overall complications, thrombosis is one of the most common associated with CVC implementation [12]. According to the literature, asymptomatic CRT accounts for up to 60% of all CRT cases [5,17].

In the present study, we reported our retrospective observations on symptomatic catheter-related thrombosis in multiple myeloma patients undergoing autologous stem cell transplantation. Literature data on the incidence of CRT in the general population ranged from 1% to 18% of patients with CVC insertion [12]. It is known that the presence of cancer increases the risk of developing CRT [18,19], especially in the advanced stages of the disease [20]. Among neoplasms, hematological malignancies seem to have similar CRT risks to solid tumors with an incidence of 1.5–18% [10,21]. Both allogeneic and autologous stem cell transplantation have a similar risk of symptomatic CRT incidence that ranges from 2.5% to 4.8% [14]. As in previous studies performed on MM patients during ASCT, symptomatic CRT was present in 2.5% of analyzed patients in our cohort [5,22]. Thus far, there are no established risk factors for CRT and no prognostic model to predict CRT during ASCT and to administer prophylaxis in the group of patients that bear the highest risk of thrombosis development with no or clinically irrelevant bleeding risk.

Despite its relative low incidence, CRT in ASCT had a severe impact on therapeutic management [23]. We currently lack guidelines both in prophylaxis and treatment of CRT during stem cell transplantation. The currently updated American Society of Hematology (ASH) guidelines for 2021 do not recommend the routine administration of parenteral or oral thromboprophylaxis for patients with cancer and a CVC [24]. Due to the risk of thrombocytopenia the leading anticoagulation therapy approach still remains low-molecular-weight heparin (LMWH). According to updated guidelines, direct oral anticoagulants (DOACs), such as apixaban or rivaroxaban, may be offered in the low-bleeding risk group and for platelet counts > 50 G/L, especially after hospital discharge [24,25,26,27]. One of the most important limitations of anticoagulant therapy is the grade 3 and 4 thrombocytopenia observed in the peri-transplant period [11]. Consistent with literature, a low platelet count does not reduce thrombotic risk [28]. On the other hand, a potential higher bleeding tendency may enforce caregivers to reduce anticoagulant dosing which may decrease anticoagulation efficacy [11]. Therefore, identification of factors increasing CRT risk is crucial for individual thrombotic risk assessment and, thus, introduction of personalized antithrombotic prophylaxis.

Patient-related factors on CRT development are not clearly defined. We also attempted to identify specific patient–related, disease-related, treatment-related, and catheter-related risk factors for CRT. In some studies, advanced age [29,30] and BMI > 25 kg/m^2^ [29,31] have been identified as factors increasing CRT risk, while other authors have not observed any impact of these factors on CRT incidence [10]. In our cohort, we did not observe any statistical significance of these factors on the CRT incidence in patients with MM during ASCT. Another considered factor was individual thrombotic predisposition. Some previous studies suggested the impact of inherited thrombophilia, such as the factors V Leiden and prothrombin mutation, on increased CRT development [21]. In the presented study, none of the patients had a history of thrombophilia. Moreover, thrombophilia testing in its current form does not significantly affect clinical management or improve outcomes for most VTE patients, particularly patients with cancer [31]. On the other hand, previous TEs were associated with a higher CRT risk (OR 2.49; 95% CI; 1.15–5.4, *p* = 0.021), moreover, this effect was dependent on the number of TEs (OR 2.75; 95% CI; 1.01–4.7, *p* = 0.047). This observation confirms other investigators’ findings about previous VTE impact on CRT risk [21,32]. Interestingly, we observed a significant impact on CRT risk for previous thrombotic episodes during initial MM treatment (OR 2.75; 95% CI; 1.15–6.53, *p* = 0.022). This may suggest that the patient’s individual thrombotic predisposition mechanism of CRT development may be related to the patient’s reaction to anticancer treatment or the disease itself. In the present study, we neither observed any influence of the M protein type nor stage of disease in the Durie–Salmon or International Staging System on CRT development. Furthermore, we did not notice any effect of disease status according to IMWG criteria before ASCT. However, it must be emphasized that almost all patients achieved remission before ASCT, and 70% achieved at least very good partial response (VGPR); thus, patients with an optimal response to previous treatment underwent ASCT. Moreover, one of the postulated factors triggering thrombotic complications is endothelial damage [12,33]. Direct irritation of the vessel wall by chemotherapy may induce local inflammation with endothelial injury and a local imbalance between pro- and anticoagulant mechanisms and, as a result, promote CRT development [34]. Among treatment-related risk factors for TE, chemotherapy is a well-known risk factor in cancer patients and increases thrombotic risk by 6.5 times. Yi and King observed the chemotherapy impact on the CRT risk in patients with PICCs [35,36]. All patients from our cohort received melphalan as a conditioning regimen with a dosing of 140–200 mg/m^2^, and we found no difference in CRT incidence between patients receiving full and reduced melphalan doses. However, in the present research, most cases of CRT were detected at median 10 days, after receiving chemotherapy, and its impact may be neglected in the pathogenesis of TEs.

Many studies have shown a relationship between catheter-related infection and CRT [5,21]. One of the proposed mechanisms is fibrin sheath formation on the catheter. The fibrin sheath, composed of various serum proteins, such as fibrin, fibronectin, and collagen, facilitates bacterial adhesion to the catheter surface [37]. Furthermore, various bacterial enzymes and endotoxins, may promote local thrombosis [21,37]. Additionally, acute inflammatory response leads to local edema and may impair blood flow through the vessel and, thus, may fulfill the third element of Virchow’s classical triad [33]. Moreover, the systemic inflammatory response increases thrombotic activity with platelet activation, the release of prothrombotic proteins such as the von Willebrand factor and the tissue factor by endothelial cells [38]. In our study, catheter-related infection was observed in 12% of cases, and increased CRT risk 2.4 times (*p* = 0.026). Most of the central venous line infections were caused by staphylococci, which confirms the observations of other authors [39,40].

It is noteworthy that the thrombotic risk may be increased by noninfectious complications as well. Previous studies have reported the influence of thrombotic risk of such complications as high leukocyte peaks after engraftment [5]. In our cohort, we observed a four times higher risk of CRT in patients with gastrointestinal complications featuring excessive vomiting and noninfectious diarrhea. Moreover, an increased risk can be explained by limited mobility of patients and possible dehydration.

We also aimed to investigate catheter-related risk factors. The first analyzed risk factor for CRT was insertion access. Based on recent literature, left-sided CVC carries a higher risk of CRT [11]. This can be explained by anatomical differences and, thus, more traumatic catheterization. Due to the limited number of CRT events analyzed in our study, we did not observe any impact of CVC access on CRT risk. This can be explained by the small group of CRT patients in our cohort. Moreover, the most common location of CVC in our patients was the right internal jugular vein.

In comparison to hematological malignancies, it has been shown that MM patients have a lower risk of CRT during ASCT than lymphoma patients (2% vs. 11%) [5]. The basis of this phenomena of lower CRT risk in the MM group remains unclear, but the shorter time from placement to removal in MM patients versus other hematological neoplasms depending on different conditioning regimens and longer neutropenic periods may be responsible for the decreased CRT risk. In our analysis, median CRT onset was 14 days after CVC insertion, and this is clinically significantly shorter than in other studies where it was 17–44 days [5,41].

Our study has several strengths. The study population was quite homogenous because we included only consecutive patients undergoing ASCT for MM within the study period. All patients were managed with the same procedure according to myeloablative regimen and supportive care management in one hospital so there were no missing data. The study population comprised only a Caucasian population so racial disparities in the risk of thrombosis can be discounted. Furthermore, we analyzed only symptomatic CRT events because there was no routine screening for TE. The presented study has several limitations that require attention. First, this study was limited by its retrospective nature and the resulting non-standardized documentation, treatment, and follow-up. Second, the retrospective character of the analysis is associated with the absence of data on potential confounding factors. Despite a relatively large group of patients with MM undergoing ASCT in comparison with previous studies, we observed a relatively low incidence of CRT cases. The small number of CRT cases may result in an underestimation of the influence of some specific factors on CRT development. Moreover, we analyzed only symptomatic CRT, and the overall incidence rate of CRT in MM patients during ASCT remains undefined.

## 5. Conclusions

In conclusion, we found that the incidence of CRT in patients with MM during CRT was relatively low (2.5%). Catheter-related infection, previous thrombotic episode before ASCT, and noninfectious complications were all associated with higher CRT incidence. The question of whether patients with MM undergoing ASCT should receive thromboprophylaxis is still open and it should be clarified in further prospective studies.

## Figures and Tables

**Table 1:** Baseline patients’ characteristics and comparisons in the groups with/or without CRT, $\bar{x\;}$ ± SD (median; Q1–Q3).

Parameter	All Cases *n* = 276	CRT Group *n* = 7	Group without CRT *n* = 269	*p*-Value
	$\bar{x}\;$**±** **SD** **(Me; Q_1_–Q_3_)**			
Age at diagnosis (Years)	55.69 ± 8.16 (57; 51–62)	58.57 ± 7.23 (62; 54–63)	55.61 ± 8.18 (57; 51–62)	0.309 ^a^
Age at ASCT (Years)	57.51 ± 8.14 (58; 53–64)	60.29 ± 7.63 (63; 56–66)	57.44 ± 8.16 (59; 53–64)	0.292 ^a^
Body weight (kg)	77.25 ± 15.42 (78; 66–87)	82.71 ± 20.41 (85; 70–95)	77.11 ± 15.29 (76.5; 66–86)	0.276 ^a^
Height (cm)	167.0 ± 10.08 (167;160–174)	168.14 ± 10.92 (174; 157–177)	166.97 ± 10.08 (166; 160–173)	0.656 ^a^
BMI (kg/m^2^)	27.57 ± 4.21 (27; 24.49–30.49)	29.06 ± 6.08 (29; 25.85–35.51)	27.54 ± 4.16 (27; 24.49–30.49)	0.357 ^a^
CCI (pts)	3.6 ± 1.00 (4; 3–4)	3.71 ± 0.95 (4; 3–4)	3.6 ± 1.00 (4; 3–4)	0.708 ^a^
HCT-CI (pts)	0.60 ± 0.87 (0; 0–1)	1.14 ± 1.22 (1; 0–2)	0.56 ± 0.85 (0; 0–1)	0.209 ^a^
Serum creatinine level (µmol/L)	74.19 ± 20.59 (71; 61–84)	75.14 ± 20.07 (66; 62–86)	74.16 ± 20.64 (71; 61–84)	0.956 ^a^
GFR MRDR (mL/min/1.73 m^2^)	99.0 ± 33.46 (94; 76–115)	92.33 ± 27.79 (89; 64–124)	99.54 ± 33.62 (94; 76- 115)	0.665 ^a^
Time to granulopoiesis recovery (days)	11.0 ± 1.11 (11; 11–12)	11.0 ± 1.0 (11; 11–12)	11.16 ± 1.12 (11; 11–12)	0.921 ^a^
Time to thrombopoiesis recovery (days)	10.0 ± 1.77 (10; 9–11)	11.14 ± 3.44 (10; 9–13)	10.23 ± 1.71 (10; 9–11)	0.821 ^a^
Hospitalization time (days)	17.0 ± 4.23 (16; 14–18)	19.57 ± 6.13 (20; 14–26)	16.67 ± 4.15 (16; 14–18)	0.181 ^a^
Observation time (days)	1196 ± 1012 (957; 454–1609)	590 ± 634 (455; 209–514)	1212 ± 1016 (977; 456–1611)	0.041 ^a^

^a^ χ^2^ test with Yates’ correction. $\bar{x}$—mean value; SD—standard deviation; Me—median; Q_1_—first quartile; Q_3_—third quartile; CRT: cancer associated thrombosis; ASCT: autologous stem cell transplantation; BMI: body mass index; CCI: Charlson comorbidity indices; HCT-CI: hematopoietic stem cell transplantation comorbidity index: GFR: glomerular filtration rate; MDRD: Modification of Diet in Renal Disease.

**Table 2:** Comparison of analyzed risk factors in patients with/or without CRT.

Parameters	All Patients % (*n* = 276)	CRT Group % (*n* = 7)	Group without CRT % (*n* = 269)	*p*-Value
Patient-Related Risk Factors				
Male gender	56.2 (155)	57.1 (4)	56.1(151)	0.739 ^a^
eGFR < 60 mL/min/1.73 m^2^	11.6 (32)	0 (0)	11.9 (32)	0.858 ^a^
TE history Other VTE Previous VTE VTE during initial MM treatment	16.7 (46) 12 (33) 5.4 (15)	42.9 (3) 42.9 (3) 28.6 (2)	16 (43) 11.2 (30) 4.8 (13)	0.171 ^a^ 0.023 ^a^ 0.022 ^a^
Disease-related risk factors				
Durie-Salmon > 2	77.2 (213)	71.4 (5)	77.3 (208)	0.370 ^b^
ISS > 2	17.9 (31)	0 (0)	18.5 (31)	0.104 ^b,c^
Prior therapy lines > 2	11.6 (32)	42.9 (3)	10.8 (29)	0.704 ^b^
Response < VGPR	30.1 (83)	42.9 (3)	29 (80)	0.886 ^b^
Treatment-related risk factors				
All complications	58.3 (161)	100 (7)	57.2 (154)	0.061 ^a^
Infectious complications	55.8 (154)	85.7 (6)	55 (148)	0.223 ^a^
Catheter-related infection	12.3 (34)	42.9 (3)	11.5 (31)	0.056 ^a^
Noninfectious complications	6.2 (17)	28.6 (2)	5.6 (15)	0.089 ^a^
Gastrointestinal complications	3.4 (9)	28.6 (2)	2.6 (7)	0.006 ^a^
Cardiac arrythmia	1.8 (5)	0 (0)	1.9 (5)	0.284 ^a^
Catheter-related risk factors				
Right sided insertion	56.9 (157)	100 (7)	55.8 (150)	0.593 ^a^
Jugular vein insertion	56.9 (157)	57.1 (4)	56.9 (153)	0.767 ^a^

^a^ χ^2^ test with Yates’ correction; ^b^ χ^2^ NW test; ^c^ available data for ISS: All cases: 168/276; CRT-group: 6/7; VTE: venous thrombotic event; MM: multiple myeloma; ISS: international staging system; VGPR: very good partial response.

**Table 3:** Multivariate analysis model determining factors that affect CRT in patients with MM undergoing ASCT.

	Odds Ratio	95% CI	*p*-Value
TE prior to ASCT	2.78	1.21–6.40	0.016
CVC-related infection	2.88	1.24–6.70	0.014
Noninfectious complications	2.75	1.10–6.91	0.031

## Data Availability

The data that support the findings of this study are available from the corresponding author, A.H., upon reasonable request. This study is a part of a larger project and some results have not yet been published.
